# The cause–effect relation of tuberculosis on incidence of diabetes mellitus

**DOI:** 10.3389/fcimb.2023.1134036

**Published:** 2023-06-26

**Authors:** Manoj Kumar Bisht, Priyanka Dahiya, Sudip Ghosh, Sangita Mukhopadhyay

**Affiliations:** ^1^ Laboratory of Molecular Cell Biology, Centre for DNA Fingerprinting and Diagnostics (CDFD), Hyderabad, India; ^2^ Regional Centre for Biotechnology, Faridabad, India; ^3^ Molecular Biology Unit, Indian Council of Medical Research (ICMR)-National Institute of Nutrition, Jamai Osmania PO, Hyderabad, India

**Keywords:** *Mycobacterium tuberculosis*, insulin resistance, diabetes, inflammation, therapeutic strategies

## Abstract

Tuberculosis (TB) is one of the oldest human diseases and is one of the major causes of mortality and morbidity across the Globe. *Mycobacterium tuberculosis* (Mtb), the causal agent of TB is one of the most successful pathogens known to mankind. Malnutrition, smoking, co-infection with other pathogens like human immunodeficiency virus (HIV), or conditions like diabetes further aggravate the tuberculosis pathogenesis. The association between type 2 diabetes mellitus (DM) and tuberculosis is well known and the immune-metabolic changes during diabetes are known to cause increased susceptibility to tuberculosis. Many epidemiological studies suggest the occurrence of hyperglycemia during active TB leading to impaired glucose tolerance and insulin resistance. However, the mechanisms underlying these effects is not well understood. In this review, we have described possible causal factors like inflammation, host metabolic changes triggered by tuberculosis that could contribute to the development of insulin resistance and type 2 diabetes. We have also discussed therapeutic management of type 2 diabetes during TB, which may help in designing future strategies to cope with TB-DM cases.

## 1 Introduction

Tuberculosis (TB) is a major health burden in the world. About 10.6 million people fell ill with TB and approximately 1.6 million deaths have occurred in the year 2021 (World Health Organization, 2022). The severity of the disease is further increased by malnutrition, poverty, smoking, alcohol abuse, and co-infection with other diseases like HIV (human immunodeficiency virus) and diabetes (Yorke et al., 2017). The association of tuberculosis with HIV and diabetes mellitus (DM), further complicates TB treatment (Baker et al., 2011; Piggott and Karakousis, 2011; Niazi and Kalra, 2012). Diabetes is a complex metabolic disorder characterized by elevated blood glucose levels due to insulin resistance, insufficient insulin production or both (Sami et al., 2017). Majority of the diabetic patients can be classified into two broad categories, type 1 and type 2. The type 1 diabetes is mostly related to autoimmune destruction of pancreatic β-cells resulting in absolute or near absolute failure of insulin production and secretion. It constitutes about 5% - 10% of total diabetic population. Whereas type 2 diabetes is related to insulin resistance (Gharravi et al., 2018; Tan et al., 2019). Uncontrolled diabetes can result in serious consequences like diabetic nephropathy (kidney dysfunction), pancreatic inflammation (leading to persistent hyperglycaemia), liver damage (cirrhosis and liver failure), inflammatory gut, and insulin resistance in liver and skeletal tissue (Daryabor et al., 2020; Selby and Taal, 2020). Tuberculosis and diabetes mellitus co-occurrence has been observed in about 0.4 million people in 2021 (World Health Organization, 2022). It is debatable whether active tuberculosis increases the risk of diabetes, however, various studies suggest that active TB can cause impaired glucose tolerance (IGT) which may lead to onset of type 2 diabetes (Mugusi et al., 1990; Oluboyo and Erasmus, 1990; Jeon et al., 2010). It is reported that impaired blood glucose tolerance can be normalized after the successful treatment of TB, but it probably persists as a risk factor for developing type 2 diabetes mellitus in the future (Yorke et al., 2017; Krishnappa et al., 2019). Several studies suggest that 5% - 30% of TB patients have concomitant diabetes mellitus and the rise in diabetic patients is highly predominant in poor and developing countries where the TB epidemic is more widespread, making TB-DM co-occurrence more frequent (Nichols, 1957; Mugusi et al., 1990; Nijland et al., 2006; Ruslami et al., 2010; Niazi and Kalra, 2012; Deng et al., 2016; Atlas IDFD, 2021; World Health Organization, 2022). India, Indonesia, China, Philippines Pakistan, Nigeria, Bangladesh, and Democratic Republic of Congo contribute to more than 2/3rd of world TB population in 2021 and are among the highest growth rate of diabetic patients (Atlas IDFD, 2021; World Health Organization, 2022). The International Diabetes Federation (IDF) has reported that the current worldwide diabetes disease burden is about 537 million, which can hit 643 million by 2030 and 783 million by 2045 (Atlas IDFD, 2021). Evidence indicating active TB disease may influence the pathogenesis of insulin resistance and incidence of type 2 diabetes (Guptan and Shah, 2000; Jeon et al., 2010; Workneh et al., 2017; Magee et al., 2018; Menon et al., 2020). Interestingly, latent TB can also influence type 2 diabetes incidents and about 25 global population have latent TB condition (Magee et al., 2022) as well as type 2 diabetes can also be a risk factor for the acquisition of latent TB infection (Lee et al., 2017; Ssekamatte et al., 2021). The combination of TB and DM (TB-DM) may interfere with the therapeutic intervention of TB as well as DM and can influence the disease course. There is a high burden of DM among TB patients at global level, however, the global prevalence of TB among DM patients is low (Workneh et al., 2017). Therefore, it is important to understand the factors that contribute to effective TB-DM therapeutic management. This review will emit light on correlation of tuberculosis and diabetes, and the possible causal factors responsible for occurrence of DM in TB patients.

## 2 TB-DM complications and DM susceptibility in TB patients

TB-DM co-occurrence may cause adverse effect on TB treatment and may subsequently increase the multi-drug resistance incidence (Baker et al., 2011; Tegegne et al., 2018). Delayed sputum conversion from smear-positive to smear-negative and higher treatment failure was observed in TB-DM patients as compared to non-diabetic TB patients (Viswanathan et al., 2014). Further studies showed that the mortality rate was higher in tuberculosis patients with DM and the MDR-TB rate was 1.6 to 3.8 folds higher in TB-DM patients as compared to TB alone (Workneh et al., 2016; Evangelista et al., 2020). However, many reports have shown that TB makes ground for diabetes or pre-diabetes conditions (Yorke et al., 2017). In most cases, these pre-diabetic symptoms turn to normal after successful tuberculosis treatment (Oluboyo and Erasmus, 1990 **)** but frequently hyperglycemia and type 2 diabetes is observed as a side-effect of TB (Magee et al., 2018). The prevalence of DM among tuberculosis patients in Ethiopia was 74 out of 1000 patients (Omar et al., 2021) and in India (second highest diabetic patients in the world [Atlas IDFD, 2021]), the prevalence of diabetes among TB patients was 25.3% and that of pre-diabetes was 24.5% (Viswanathan et al., 2012). The prevalence of diabetes mellitus among TB patients in South India was shown to be 39%, indicating one in three TB patients had co-existing DM (Rajaa et al., 2021). TB epigenetically enhances DM-linked complication pathways (Prada-Medina et al., 2017). Thus, it is very important to understand the factors/mechanisms that are responsible for occurrence of DM in TB patients (Atlas IDFD, 2021; World Health Organization, 2022). Various factors and cellular signaling cascades that can lead to the development of diabetic/pre-diabetic conditions in TB patients are described below.

### 2.1 Insulin resistance

Insulin regulates blood glucose levels through glucose and lipid metabolism in the body. Insulin resistance is clinically defined as a condition where known amount of exogenous or endogenous insulin fails to increase glucose uptake and utilization as much as in a normal population (Lebovitz, 2001). In other words, the body develops diminished response to the actions of insulin or impaired sensitivity to insulin resulting in higher blood glucose levels. Insulin resistance is one of the pre-diabetic conditions that can lead to diabetes and metabolic syndromes (Suzuki et al., 1996; Ormazabal et al., 2018). IR is often associated with tuberculosis and is suggested to be a risk factor as well as a potential marker for active tuberculosis (Mao et al., 2011, Yurt et al., 2013). A cross-section cohort study in South Africa showed correlation between TB and IR in newly diagnosed TB patients which gets reverted after successful treatment (Philips et al., 2017). The overall (both male and female) prevalence of IR in this study was 25.4% (Philips et al., 2017). It has been shown that Mtb-infected mice are more susceptible to develop hyperglycaemia and insulin resistance than uninfected mice and this susceptibility is further increased with high-fat diet and age (Oswal et al., 2022). Mtb infection causes dysregulation of lipid metabolism and elevates the levels of circulating free fatty acids which causes ectopic deposition of lipids in organs important for glucose homeostasis like liver and skeletal muscles leading to development of IR (Badin et al., 2011; Oswal et al., 2022). Multiple studies suggest that TB-DM/IR incidents occur with altered lipid metabolism that includes high LDL (low-density lipoprotein) cholesterol, low HDL (high-density lipoprotein) cholesterol, high levels of very low-density lipoprotein (VLDL) triglycerides, resulting in severe clinical manifestation and disease outcome (Vrieling et al., 2018; Shvets et al., 2021). IR was also observed during the early phase of anti-tuberculosis therapy which can be attributed to impaired liver function due to the toxic effect of first-line anti-tuberculosis drugs like isoniazid, rifampicin and pyrazinamide (Shvets et al., 2019). Insulin resistance results in abnormalities in PI3K/Akt pathway causing wasting in muscle tissue which is also observed in tuberculosis patients (Wang et al., 2006; Mupere et al., 2014).

### 2.2 Hyperglycaemia

Tuberculosis patients generally have temporal hyperglycaemic conditions. Among the patients that are hyperglycaemic during the start of TB treatment, a high proportion of patients receive a new diabetes diagnosis without prior DM history **(** Magee et al., 2018). The incidence of hyperglycaemia in tuberculosis may be attributed to stress, prolonged inflammation, change in the glucose and lipid metabolism of TB patients and insulin resistance syndrome (Guptan and Shah, 2000; Magee et al., 2018; Menon et al., 2020). A clinical study in India suggests that 7% tuberculosis patients had DM and 4.5% patients have IGT or impaired blood glucose tolerance and about 65% of IGT patients could revert to normoglycemic conditions after completion of anti-TB therapy (Krishnappa et al., 2019). There is a study in Periurban South Africa that shows an association between transient hyperglycemia, and TB-DM/TB-IGR (impaired glucose regulation) in newly diagnosed DM patients as well as an association between persistent hyperglycemia and TB-DM in patients with HIV-1, despite TB therapy (Kubjane et al., 2020). A meta-analysis investigation showed that 27.3% TB patients had newly developed hyperglycaemic condition at baseline level and after a follow-up of 3 - 6 months, 50% of the hyperglycaemic conditions remained unresolved (Menon et al., 2020). In a follow-up study in Brazil with HIV-positive TB patients, Moreira et al. (2018) had indicated that hyperglycemia frequently occurs in HIV-infected patients who initiate TB treatment, and it increases the risks of adverse TB outcomes (Moreira et al., 2018). Another screening study for diabetes among TB patients in India showed the prevalence of newly diagnosed diabetes was approximately 10.8% where HbA1c ≥ 47.5 mmol/mol was used for diagnosis of diabetes (Kumpatla et al., 2013). Various studies reveal that the prevalence of hyperglycaemia in tuberculosis patients varies between 10% to 26% which depends on age, sex and fasting blood glucose level (Moreira et al., 2018; Menon et al., 2020; Ephraim et al., 2021). An interesting study indicated that hyperglycaemia is associated with increased risk of patient delay in pulmonary tuberculosis which is a serious concern in the context of diagnosis of TB, increased risk of transmission of TB infection in the community as well as clinical manifestation of TB (Wang et al., 2017). Liver toxicity during tuberculosis is often associated with the first line anti-TB drugs which increase hepatic enzyme levels and may be responsible for hyperglycemia in diabetic patients (Molla et al., 2021). TB-DM and TB patients have high levels of liver enzymes compared to normal (Sama et al., 2017). The hyperglycemic response against anti-TB drugs like rifampicin may enhance the requirement for insulin in type 1 diabetes patients (Atkin et al., 1993). Thus, rifampicin disturbs glycemic control in both type 1 and type 2 diabetes.

### 2.3 Inflammation

Inflammation is critical for controlling infection and a dysregulation inflammatory response is central to pathogenesis of several pathogens including tuberculosis. The ability of *M. tuberculosis* to modulate the fundamental inflammatory processes such as recruitment of immune cells to the site of infection, prodcution of pro-inflammatory cytokines, production of eicosanoids and lipid mediators associated with inflammation favors its survival inside the host (Kaufmann and Dorhoi, 2013). Inflammation during active tuberculosis results in an environment with higher pro-inflammatory cytokine production as a protective response in host against the bacilli. Inflammation is nonresolving in nature in both active and latent tuberculosis (Kaufmann and Dorhoi, 2013), characterized by elevated levels of pro-inflammatory cytokines (He et al., 2020) which often lead to metabolic dysregulation and eventually type 2 diabetes (Spranger et al., 2003; Das and Mukhopadhyay, 2011; Tsalamandris et al., 2019). On the other hand, the hyperglycaemic environment often results in dysfunction of the immune system like inhibition of leukocyte recruitment, damage to the neutrophil function, macrophage dysfunction, depression of the antioxidant system and humoral immunity as well as modulation of inflammatory signaling cascades causing the DM patients to be susceptible to various infections (Casqueiro et al., 2012; Berbudi et al., 2020).

Co-incidence of pulmonary TB with pre-diabetes causes elevated type-I, type-II, and Th17 cytokine responses with higher circulating levels of interferon-gamma (IFN-γ), IFN-β, tumor necrosis factor-alpha (TNF-α), interleukin (IL)-2, IL-5, IL-17A, IL-17F, IL-10, IL-1β, IFN-β, transforming growth factor-β (TGF-β) and Granulocyte-macrophage colony-stimulating factor (GM-CSF), however, no significant correlation was observed between Mtb bacterial burden and cytokine levels (Kumar et al., 2014).

Toll-like receptors (TLRs) are members of the innate immunity that works as pathogen-associated molecular pattern (PAMP) receptors and play a crucial role in eliciting innate immune response which dictate subsequent adaptive immune responses (Akira and Takeda, 2004). Activation of TLRs usually triggers a signaling cascade which results in the production of pro-inflammatory cytokines/chemokines through nuclear factor kappa B (NF-κB) (Udgata et al., 2019). Several mycobacterial components are recognized by TLR2 and TLR4 (Means et al., 1999; Heldwein and Fenton, 2002). Several lines of evidences point to a role of TLRs in the development of insulin resistance and diabetes. Among the TLRs, TLR2 and TLR4 expression have been shown to be increased in conventional insulin resistance target tissues like skeletal muscle and adipose tissue of type 2 diabetic subjects (Creely et al., 2007; Reyna et al., 2008). Song et al. (2006) showed that TLR4 triggering leads to NF-κB activation and expression of TNF-α, IL-6, and resistin which have been implicated for the development of insulin resistance in adipocytes. Type 2 diabetic subjects were found to have significantly increased TLR2, TLR4 mRNA, and protein in monocytes and increased TLR2 and TLR4 expression was correlated with BMI, homeostasis model assessment–insulin resistance (HOMA-IR), glucose, A1C, Nϵ-(carboxymethyl) lysine (CML), and free fatty acid (FFA) (Dasu et al., 2010). Nonetheless, the expression levels of TLR2 were found to be upregulated in obese individuals who are at higher risk of development of insulin resistance as obese type 2 diabetes patients had higher expressions of TLR2 in comparison to obese patients without type 2 diabetes (Ahmad et al., 2012).

Interestingly, signaling through TLR2 can elicit both pro- and anti-inflammatory response depending on the nature and site of interaction with its ligand on the leucine rich repeat (LRR) motif present on the ectodomain of TLR molecule (Udgata et al., 2019). It has been found that a member of the PPE family of Mycobacterium tuberculosis, PPE18 interact with LRR 11~15 on TLR2 ectodomain and induces IL-10 production from macrophages and elicits anti-inflammatory responses (Nair et al., 2009). In contrast, PPE17 protein of Mtb was found to specifically interact with TLR2 LRR 15~20 domain to activate NF-κB and induced pro-inflammatory-type signaling (Bhat et al., 2012). Also, it has been shown that the cellular localization of heat shock protein 60 of Mtb (Mtbhsp60) upon interaction with TLR4 can dictate the inflammatory responses in macrophages (Parveen et al., 2013). Thus, there is a possibility of induction of insulin resistance by Mtb using PPE17/Mtbhsp60 by targeting the TLR-NF-κB-triggered signaling cascades. Interestingly, it has been shown that *Mycobacterium* sp. utilizes TLR2 to promote inflammation (Kanagawa et al., 2015). Mycobacterial secretory protein MTP53 (Rv2878c), a disulphide bond-like forming protein also induces TGF-β-activated kinase 1-mediated pro-inflammatory cytokine response during Mtb infection (Wang et al., 2019; Pal et al., 2022). Some lipases of Mtb can also trigger inflammatory responses and modulate immune responses (Rameshwaram et al., 2018). For example, Rv0183 is involved in triggering MyD88-NF-κB signaling and induction of inflammatory molecules like IL-6, TLR2, TLR6 and TNF-α (Xu et al., 2010). Another lipase LipC can hydrolyze short-chain esters and elicits cytokines/chemokines induction (Shen et al., 2012). The lipases have an adverse effect on patients, as they cause the production of inflammatory cytokines as well as generates excess free fatty acids/lipid mediators. Excess free fatty acids get deposited in tissues important for glucose homeostasis like liver and muscle to cause systemic insulin resistance that may lead to insulin resistance (Martinez et al., 2019).

Inflammation also plays a key role in the development of insulin resistance and T2D during obesity as deletion of jun kinase 1 (JNK1, a key negative regulator of insulin sensitivity in the obese state) in the nonhematopoietic compartment protects mice against HFD-induced insulin resistance and T2D by inhibiting HFD-induced inflammation (Solinas et al, 2007; Das and Mukhopadhyay, 2011). Inflammation changes the expression of key genes of adipose tissue including SREBP-1, Resistin, RANTES, leptin, and adiponectin involved in glucose homeostasis. The sterol regulatory element binding protein-1 (SREBP-1) regulates genes that are involved in fatty acid synthesis and lipogenesis during inflammation. TNF-α completely blocks SREBP-1 expression and its activation (Sewter et al., 2002; Das and Mukhopadhyay, 2011). Resistin also links to inflammation and higher resistin levels were observed in tuberculosis and T2D patients (Youn et al., 2004; Gharibeh et al., 2010; Das and Mukhopadhyay, 2011; Moideen et al., 2018). Resistin activates pro-inflammatory cytokines like IL-12 and TNF-α through the NF-κB-dependent pathway in mouse and human macrophages and is directly correlated to glucose homeostasis (Asensio et al., 2004; Silswal et al., 2005). RANTES also known as CCL5 is an adipokine secreted by adipocytes and involved in the recruitment of macrophages, T cells, eosinophils, and basophils (Appay and Rowland-Jones, 2001; Keophiphath et al., 2010) and cause inflammation. RANTES also impaired glucose-induced insulin secretion in mice by reducing the glucose-dependent secretion of glucagon-like peptides (Pais et al., 2014). RANTES is also involved in insulin responsiveness through CCR5, which regulates insulin signaling in the hypothalamus and contributes to systemic insulin sensitivity and glucose metabolism (Chou et al., 2016).

### 2.4 Adipose tissue

Adipose tissue functions as an endocrine organ by regulating metabolism and inflammation through secretion of adipokines, cytokines, peptides, and release of free fatty acids. Reports indicate that adipose tissue inflammation is consistently associated with excess fat mass and insulin resistance and development of type 2 diabetes (Guilherme et al., 2008; Burhans et al., 2019). Mtb infection in adipose tissue causes adipocyte hypertrophy, lipolysis, alterations in metabolic processes, and infiltration of immune cells (Ayyappan et al., 2019). Hypertrophic or enlarged adipocytes are characterized by altered secretion patterns of adipokines, as well as secretion of higher levels of pro-inflammatory cytokine like TNF-α and MCP-1, which not only impair insulin signaling in the skeletal muscle and liver but also induce systemic insulin resistance (Guilherme et al., 2008). Adipose tissue produces chemokine monocyte chemoattractant protein-1 (MCP-1/CCL2) and leptin which are strongly associated with inflammation. The adipokine MCP-1 promotes inflammation and diabetic nephropathy (Tesch, 2008). MCP-1 production is higher in adipose tissue and CD14^+^ monocytes in TB patients. MCP-1 is a potent pro-inflammatory molecule that plays a critical role in inducing inflammatory response during TB by attracting monocytes and T lymphocyte (Lin et al., 1998; Martinez et al., 2019). MCP-1 overexpression causes insulin resistance in adipose tissue and steatosis in the liver during obesity. Mtb-infected visceral adipose tissue has increased MCP-1 expression which alters adipocyte function (Erol, 2008). It is suggested that MCP-1 probably induces adipocyte dedifferentiation and adds to pathologies associated with hyperinsulinemia, insulin resistance and obesity, including type 2 diabetes (Sartipy and Loskutoff, 2003; Erol, 2008; Panee, 2012). Interestingly, it has been noted that addition of MCP-1 to differentiated adipocytes *in vitro* results in downregulation of insulin-stimulated glucose uptake as well as expression of various genes like LpL, adipsin, GLUT-4, aP2, beta3-adrenergic receptor and peroxisome proliferator-activated receptor gamma (PPAR-γ) (Sartipy and Loskutoff, 2003).

**Figure 1:**
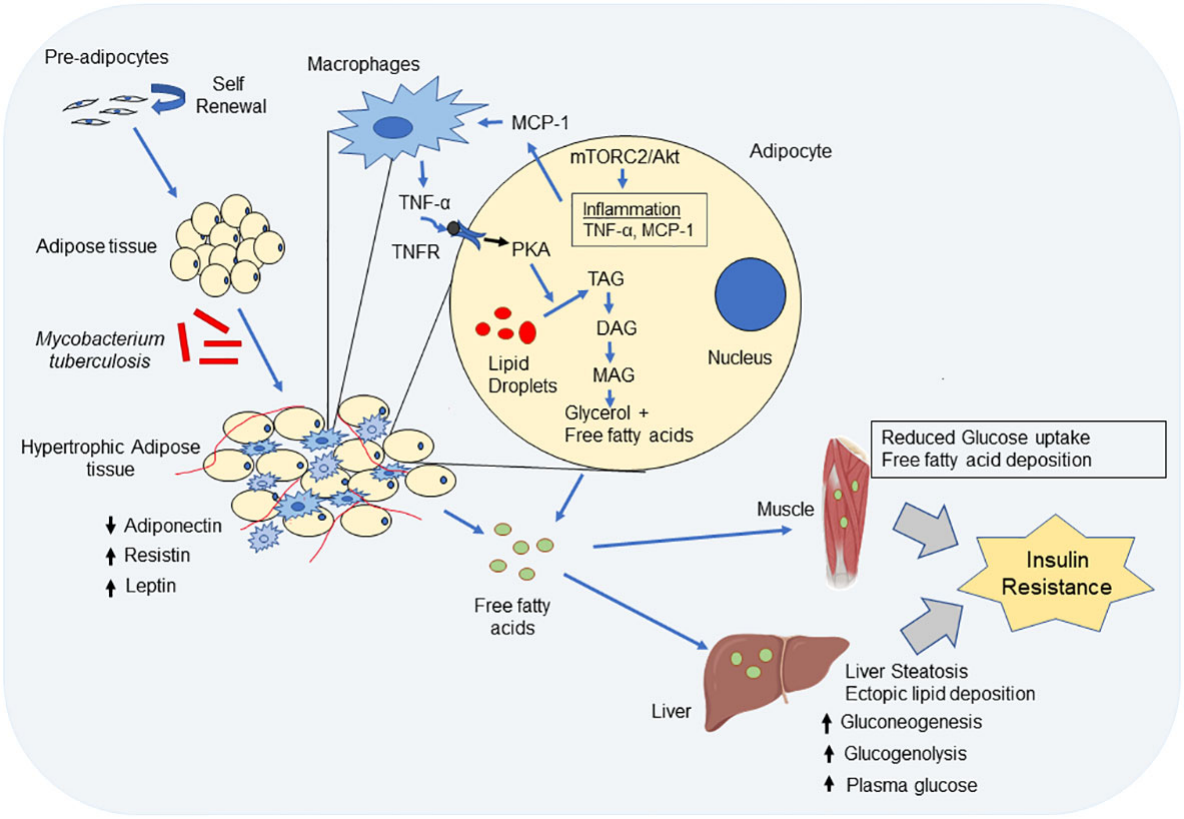
Impact of *Mycobacterium tuberculosis* (Mtb) infection on the physiological functions of adipocyte. Mtb infection leads to hypertrophy and immune cell infiltration into adipose tissue. Hypertrophic adipose tissue decreases expression of adiponectin, but increases expression of leptin and resistin. Decreased adiponectin expression reduces insulin sensitivity and through simultaneous upregulation of leptin and resistin, there is induction of inflammation and insulin resistance. Again, through mTORC2/Akt activation in adipocytes, Mtb bacteria cause inflammation and induce TNF-α and monocyte chemoattractant factor-1 (MCP-1) and reduce adiponectin. MCP-1 recruits macrophages and other cell type into adipose tissue. The infiltrated immune cells in adipocyte further produce inflammatory cytokine like TNF-α and IL-6. TNF-α binds to TNF receptor on adipocyte and induce activation of protein kinase A which phosphorylates hormone sensitive lipase and causes lipolysis in adipocytes. The inflammation in adipose tissue increased lipolysis, i.e. degradation of tri-acylglycerol (TAG) to di-acyleglycerol (DAG), mono-acylglycerol (MAG) and finally glycerol and release of free fatty acids (FFA). FFA through circulation gets deposited in other sites like liver and muscle. In liver, fat deposition leads to increase gluconeogenesis, gluconeolysis and leads to liver steatosis which results in increased plasma glucose concentration and finally leads to insulin resistance. Free fatty acid deposition in muscle tissue also causes insulin resistance in muscles.

Mtb-infected adipose tissue recruits myeloid and NK-cell lineage which produce higher levels of TNF-α, IL-12 and other inflammatory molecules (Erol, 2008; Beigier-Bompadre et al., 2017). TNF-α binds to TNFR on adipocytes and increase production of intracellular cAMP that activates protein kinase A-induced lipolysis and generates free fatty acids (Zhang et al., 2002; Song et al., 2006). The lipolytic activity of TNF-α is shown to be influenced by glucose in adipocytes (Green et al., 2004). The increased free fatty acids released by the adipocytes are ectopically deposited in various organs like liver, skeletal muscles, pancreas and gastrointestinal tract causing lipotoxicity (Sears and Perry, 2015). In liver, free fatty acid increases TAG formation/accumulation (liver steatosis), increase hepatic glucose production (increased gluconeogenesis and glucogenolysis) and insulin resistance leading to hyperglycemia (Boden, 2003; Sears and Perry, 2015). Free fatty acid accumulation in skeletal muscles inhibit glucose uptake, reduce fatty acid oxidation and reduce insulin sensitivity which, results in insulin resistance (Delarue and Magnan, 2007; Lyons et al., 2016). The physiological changes and modulation in the signaling cascades in the adipose tissue during Mtb infection that can lead to insulin resistance is described in **Figure 1**.

*Mycobacterium tuberculosis* can activate macrophages through various mechanisms like binding of pathogen associated molecular patterns (PAMPs) to the Toll like receptors (TLRs) present on the macrophages to induce production of pro-inflammatory cytokines (Dolasia et al., 2018), production of reactive oxygen species (ROS) by NADPH oxidase complex and reactive nitrogen intermediates (RNI) (Nathan and Shiloh, 2000), modulation of signaling pathways such as MAPK, NF-κB pathway (Cambier et al., 2014; Mayer-Barber and Sher, 2015). These activated macrophages can secrete various pro-inflammatory cytokines like TNF-α, IL-6 (Lumeng and Saltiel, 2011) which can lead to extensive remodeling of the adipose tissue (Sun et al., 2011; Hotamisiligil, 2017). One mechanism by which macrophage activation may contribute to adipocyte hypertrophy is through the secretion of matrix metalloproteinases (MMPs) which degrade extracellular matrix components, allowing adipocytes to expand in size (Lijnen et al., 2002). Macrophages can secrete MMPs, particularly MM2 and MMP-9, in response to *M. tuberculosis* infection (Quiding-Järbrink et al., 2001). On the other hand, levels of adipokines like resistin were found to be increased in pulmonary TB patients (Chang et al., 2013). Resistin is known to increase lipolysis in the visceral adipose tissue (Chen et al., 2014) by increasing expression of hormone sensitive lipase (HSL) leading to increased levels of serum free fatty acids (Qatanani et al., 2009) which cause a positive feedback loop that may contributes to the development of adipocyte hypertrophy (Pérez-Torres et al., 2019).Leptin is another important adipose-derived hormone, which regulates food intake and body weight, and is expressed by adipocytes of white adipose tissue (Kelesidis et al., 2010). Lower body fat and wasting in TB patients is probably associated with lower serum leptin concentrations and a gradual increase in leptin levels is observed post-TB treatment (vanCrevel et al., 2002). Also prolonged inflammation during tuberculosis may be responsible for decreased leptin production (vanCrevel et al., 2002). Leptin deficiency is shown to cause severe insulin resistance and related endocrine disorders in uncontrolled insulin-deficient diabetes (German et al., 2010).

### 2.5 Lipids and free fatty acids

Lipids and their mediators are important components of innate immunity against *M. tuberculosis* (Chen et al., 2008). The modulation in lipid profile can cause atherosclerosis, insulin resistance (Laakso et al., 1990) and other cardiovascular diseases (Dong et al., 2021). Mtb persists inside adipose tissue and alters its metabolic properties to modulate fatty acid metabolism (Neyrolles et al., 2006; Erol, 2008). Lipolysis in adipose tissue is another effect of tuberculosis that causes generation of low HDL and higher levels of total cholesterol, LDL, very low-density lipoprotein and non-esterified serum free fatty acids (NEFA) (Vrieling et al., 2018; Ayyappan et al., 2019). High serum triglyceride levels and free fatty acids result in the deposition of lipid granules in various organs, that is known to inhibit insulin-associated signaling cascades resulting in insulin resistance (Boden and Shulman, 2002; Xin et al., 2019). Lipid profile abnormalities and an increased risk of diabetes were observed in multidrug-resistant (MDR) TB patients (Biranu et al., 2021). Lipid mediator, lipoxin and leukotriene derived from arachidonic acid is associated with Mtb susceptibility and disease pathogenicity (Mayer-Barber and Sher, 2015). Interestingly, it has been observed that leukotriene B4 plays a central role in systemic inflammation and establishment of insulin resistance in animal model of diabetes (Li et al., 2015). Secretory mycobacterial lipase Rv0183 is shown to be involved in degradation of lipid bodies present in adipocyte or foamy macrophages causing generation of free fatty acids which may be responsible for metabolic syndrome during tuberculosis (Erol, 2008; Dhouib et al., 2010; Dedieu et al., 2013).

Persistent chronic inflammation is considered one of the major risk factors for development of cardiovascular diseases (Ferrucci and Fabbri, 2018) and systemic inflammation levels have been associated with sputum bacterial load (Mesquita et al., 2016). Interestingly, tuberculosis and cardiovascular diseases were found to have a close epidemiological connection (Huaman et al., 2015). It has been found that granuloma formation during tuberculosis can cause coronary arteritis leading to myocardial infarction (Rodríguez et al., 2012). In addition, production of pro-inflammatory cytokines by activated macrophages and CD4^+^ T cells can also contribute to development of cardiovascular complications (Huaman et al., 2015). The hallmarks of atherosclerosis are LDL oxidation, foam cell formation and inflammation (Chistiakov et al., 2017). Tuberculosis patients have been shown to have higher susceptibility to LDL oxidation and lipid peroxidation (Nezami et al., 2011) leading to formation of foam cell macrophages (Agarwal et al., 2021) which have been implicated to play a role in the development of TB granulomas and persisting Mtb infection. Diabetes is also often associated with dyslipidemia characterized by increased VLDL, LDL-cholesterol (LDL-C) and decreased high-density lipoprotein cholesterol (HDL-C) (Schofield et al., 2016) and the levels of oxidized LDL (oxLDL) were found to be significantly higher in diabetic patients (Banerjee et al., 2019). Uptake of oxLDL mediated by scavenger receptor CD36 accounts for a large proportion of the formation of macrophage foam cells and formation of atherosclerotic plaques (Luo et al., 2017). Furthermore, patients with concurrent TB and diabetes have been shown to have pro-atherogenic plasma lipid profile with significantly elevated levels of sphingomyelins and remnant-like lipoprotein particles (Vrieling et al., 2018). Therefore, *M. tuberculosis* infection and diabetes can independently contribute to the development of atherosclerosis.

### 2.6 Host metabolic changes

**Figure 2:**
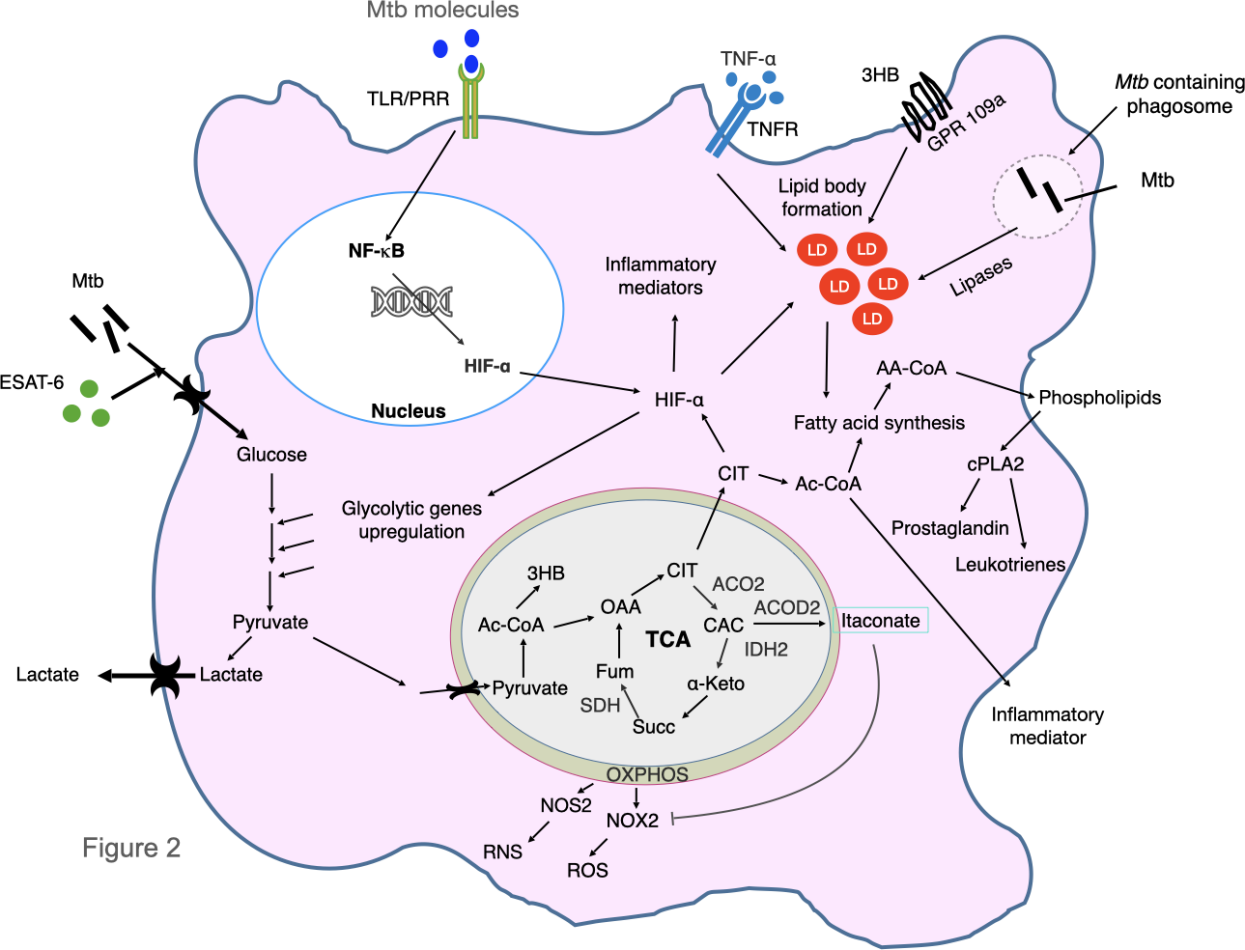
*Mycobacterium tuberculosis* infection alters host metabolic pathways. Mtb and ESAT-6 protein increase glucose uptake and up-regulate glycolytic genes and inhibit oxidative phosphorylation causing Warburg effect. This results in production of pyruvate which is converted to lactate and is secreted out, excess pyruvate is transported to mitochondria and form acetyl-CoA (Ac-CoA) and finally produce ketone bodies 3 hydroxybutyrate (3HB). 3HB binds to GPR109a and cause lipid body formation. Ac-CoA is converted to OAA and enters in the TCA (Tricarboxylic acid) cycle. The OXPHOS and TCA cycle enzymes are downregulated (that include ACO2, IDH2, ACOD2 and SDH) by Mtb. As a result, CIT is accumulated which stabilizes and increases expression of hypoxia inducible factor-α (HIF-α) and increases production of itaconate. HIF-α induce lipid body formation, upregulation of glycolysis genes and inflammatory mediator like IL-1β. Itaconate inhibits production of reactive oxygen species (ROS). Decreased OXPHOS also causes production of ROS and reactive nitrogen species (RNS). Excess CIT is converted into Ac-CoA which is utilized in the fatty acid synthesis pathway and causes formation of membrane phospholipids. Cytosolic phospholipase A2 (cPLA2) release Arachidonic acid into cytosol which gives rise to prostaglandin and leucotrienes which cause inflammation. Various toll like receptor (TLR) and pattern recognition receptor (PRR)-triggered signaling stimulation cause NF-κB activation and transcription of inflammatory mediators, as well as production of HIF-1α. *M. tuberculosis* in phagosome secrete various lipases which degrade host lipids that are responsible for generation of fatty acids. The TNF-α-mediated signaling is also involved in formation of lipid droplets. CAC, cis-aconitate; CIT, citrate; FUM, fumarate; α-KG, α-ketoglutarate; NOS2, nitric oxide synthase 2; NOX2, NADPH oxidase; OAA, oxaloacetate; PGE2, prostaglandin E2; SUCC, succinate; TNF, tumor necrosis factor; TNFR, tumor necrosis factor receptor; ACOD2, aconite dehydrogenase, ACO2, aconitase2, SDH succinate dehydrogenase, IDH2, isocitrate dehydrogenase 2, LD, lipid droplets, OXPHOS, oxidative phosphorylation, AA-CoA, acyl Co-A.

Mtb proteins can affect host glucose and lipid metabolism to create a favourable condition for the survival of the bacilli. It is reported that pre-diabetic condition is favourable for survival of Mtb bacilli (Sinha et al., 2021). Some Mtb proteins are shown to modulate host metabolism to induce a favourable niche. For example, ESAT-6 protein of Mtb induces GLUT-1-mediated glucose uptake in macrophages which perturbs glycolytic pathway and causes foamy macrophages formation (Singh et al., 2015). Interestingly, Mtb-infected macrophages have increased glucose uptake, upregulated expression of glycolytic enzymes and downregulated levels of enzymes of tricarboxylic cycle and oxidative phosphorylation which results in lactate production causing the Warburg effect. (Shi et al., 2015; Howard and Khader, 2020). This results in decreased rate of mitochondrial respiration which causes inflammation in macrophages and adipocytes which is likely to further contribute to subsequent systemic insulin resistance (Wang et al., 2020). Mtb can utilize lactate with L-lactate dehydrogenase gene, lldD2 (Rv1872c) and facilitate its intracellular survival. It has been shown that lldD2 knock-out Mtb fails to efficiently replicate in human macrophages (Billig et al., 2017). The tuberculosis necrotizing toxin (TNT) of Mtb hydrolyses NAD^+^ and causes generation of reactive oxygen species (ROS) which is a risk factor for type 2 DM (Pajuelo et al., 2020). In M1-polarized macrophages, the TLR/PRR-triggered signaling causes NF-κB-induced inflammation and production of hypoxia mediated factor-1α (HIF-1α) (Corcoran and O'Neill, 2016; Dorrington and Fraser, 2019). HIF-1α induces lipid body formation, upregulation of glycolytic genes and inflammatory mediator like IL-1β (Howard and Khader, 2020). Increased glycolysis causes pyruvate production which is transported into the mitochondria and forms 3 hydroxybutyrate and enters into tricarboxylic acid (TCA) cycle and causes accumulation of citrate which is transported to cytosol. Citrate stabilizes HIF-1α and is converted to Acetyl-CoA which fed into fatty acid biosynthesis. The acyl-CoA synthase long-chain family members, mainly ACSL1 and ACSL4 introduce long chain fatty acid especially arachidonic acid into membrane phospholipid (Shi et al., 2019). Cytosolic phospholipase A2 (cPLA2) release arachidonic acid from membrane phospholipids. Arachidonic acid gives rise to prostaglandin and leukotrienes which are responsible factors of inflammation. Mtb also secretes lipases that are involved in formation of lipid droplets which are risk factors for DM (Rameshwaram et al., 2018). For example, Rv2672c exhibits both lipase and proteinase activity (Singh et al., 2017). The other secretory mycobacterial Rv1083 protein is a monoacylglycerol lipase which is involved in metabolizing the host cell membrane lipids (Saravanan et al., 2012). Mtb Rv0183 produces glycerol and free fatty acids which further causes metabolic perturbations (Grininger et al., 2021). Rv1076 is another Mtb secretory esterase that hydrolyzes short carbon chain substrate (Kaur et al., 2017; Li et al., 2017). Other secretary enzymes like Rv1984c hydrolyzes medium chain carboxylic esters and monoacylglycerol whereas Rv3452 is a phospholipase A2 (Schué et al., 2010). Intracellular Mtb in macrophages can utilize host amino acids directly from cytosol for biosynthesis of its proteins (Borah et al., 2019). The metabolic reprogramming and wasting syndrome during tuberculosis follow conserved mechanism in human patients, mice and zebrafish. The concentration of circulating small amino acids is lower in human patients as well as mycobacterium-infected mice and zebrafish (Ding et al., 2020). Circulating amino acids like histidine, tryptophan, methionine, and glutamine are significantly decreased in TB patients but in case of TB-DM, other amino acids like choline, glycine, serine, threonine levels are also lower as compared to patients with TB alone (Weiner et al., 2012; Weiner et al., 2018; Vrieling et al., 2019). Histidine, glutamine (and the (E,E)-isomer of bilirubin) are shown to be negatively associated with development of type 2 diabetes and histidine-mediated suppression of hepatic glucose production is a potential target for the treatment of type 2 diabetes (Kimura et al., 2013; Peddinti et al., 2017). TNF-α secreted during Mtb infection also plays a major role to promote lipid droplet formation (Guerrini et al., 2018; Jung et al., 2020). The alteration of host metabolic processes during infection with Mtb is described in **Figure 2**.

In conclusion, active TB itself can induce various immune-metabolic changes like increased inflammation, adipose tissue modulation, increased free fatty acid levels leading to development of insulin resistance in patients that can lead to development of type 2 diabetes if not managed clinically. Irregular lipolysis in adipose tissue, altered glucose and lipid metabolism in adipose tissue and other organs including lungs due to increased accumulation of intracellular lipids as well as inflammatory milieu around the adipose tissue are possible reasons for development of IR during tuberculosis (Oswal et al., 2022). However, further studies are needed to find out the exact molecular mechanism of development of IR in TB patients. Prolonged hyperglycaemia can be considered as a risk factor for DM and cardiovascular diseases. The host immune response to active TB disease results in a prolonged state of systemic inflammation characterized by activation of various immune cells by binding with PAMPs through the TLRs resulting in elevated levels of pro-inflammatory cytokines like IL-1β and IL-6 which are known to directly inhibit insulin-stimulated glucose uptake and thus implicated in the etiology of type 2 diabetes. Various proteins/lipases of Mtb are involved in the activation of immune cells and thus involved in triggering inflammatory responses in the host. Modulation of adipose tissue signaling during tuberculosis may be one of the reasons of metabolic disorder and pre-diabetic conditions during tuberculosis. Adipose tissue infection by Mtb causes tissue inflammation, immune cell infiltration and modulation of adipokine secretion which altogether may be involved in generation of pre-diabetic conditions. Loss of adipose tissue due to increased lipolysis during tuberculosis leads to release of free fatty acids which in turn disrupts insulin signaling pathway in skeletal muscles and can cause systemic insulin resistance. Various Mtb proteins are responsible for host metabolic perturbation and modulation of lipid metabolism. Again, reduction in circulating amino acid levels like histidine, tryptophan, methionine and glutamine may diminish host capacity to resists pre-diabetic changes. A detail study on the molecular mechanisms by which *M. tuberculosis* disrupts host glucose homeostasis and understanding the signaling cascades is important to develop appropriate therapeutics to tackle hyperglycemic condition during TB.

## 3 Impact of diabetes on the susceptibility to tuberculosis

As a chronic condition diabetes compromises the immune system which reduce the host’s ability to fight infections. It has been found that, diabetes can cause impairment of cytokine production, inhibit leukocyte recruitment, dysfunction of immune cells like neutrophil, macrophages, and natural killer cells. In addition to dysfunction of the cellular immunity, it can affect complement effector system and antibody production (Berbudi et al., 2020). In fact diabetes increases susceptibility to various infections like HIV (Grinspoon, 2009), fungal infections (Lao et al., 2020), mycobacterium (Kumar and Babu 2017; Navid et al., 2020) and recently COVID-19 (Erener, 2020). Type 2 diabetes is considered to be a major risk factor for increased susceptibility to TB infection and reactivation (Baker et al., 2011; Yorke et al., 2017; Mukhtar and Butt, 2018; Ayelign et al., 2019; Alim et al., 2020). Diabetic individuals were found to be three times more susceptible to TB (Jeon and Murray, 2008) and also have higher risk of death during TB treatment (Gautam et al., 2021), poor TB treatment outcomes and higher risk of developing latent TB (Restrepo, 2016; vanCrevel and Critchley, 2021). Conditions which complicate TB pathology, outcome of treatment and mortality are age, poorer immune activity, inflammation, hyperglycemia, liver and chronic kidney disease which are often associated with diabetes (Alim et al., 2020; Kaur et al., 2021).

The major immune mechanisms by which diabetic patients become more susceptible to TB include but not limited to defects in bacterial recognition, reduced phagocytic activity, the slow migration rate of macrophages and other antigen-presenting cells, secretion of chemokines/cytokines and impaired T cell response that ultimately results in compromised immune responses causing increased Mtb load and disease pathogenicity in various organs like lungs and liver (Vallerskog et al., 2010; Kumar et al., 2017b; Alim et al., 2020). It has been found that the function of neutrophils, macrophages, dendritic cells, NK cells and other components of innate immunity is drastically compromised by metabolic alterations (Martinez and Kornfeld, 2014). Alveolar macrophages from chronically hyperglycemic mice were found to have impaired recognition of *M. tuberculosis* due to reduced expression of CD14 and MARCO which are involved in recognition of mycobacterial cell wall components (Martinez et al., 2016). In addition, peripheral monocytes from diabetic patients were found to have reduced capacity to bind or engulf Mtb bacilli compared to those from euglycemic controls due to modifications in the complement pathway of opsonization (Gomez et al., 2013).

Among the phagocytic cells predominantly, the neutrophils and macrophages are primarily responsible for the induction of inflammatory response during tuberculosis and have a detrimental effect on TB-DM pathogenesis (Das and Mukhopadhyay, 2011; Mishra et al., 2017; Borkute et al., 2021). Neutrophils harboring *M. tuberculosis* are abundant in inflammatory lesions and associated with lung pathology and TB disease severity (Muefong and Sutherland, 2020). Neutrophil counts are higher during TB infection and TB-DM further increases neutrophil count (Prada-Medina et al., 2017). Higher neutrophil leukocyte ratio is a potential marker of inflammation and can cause TB disease severity (Abakay et al., 2015), insulin resistance and type 2 diabetes (Guo et al., 2015; Lou et al., 2015). Tissue-specific inflammation caused by Mtb infection can also cause modulation of signaling mechanisms and metabolic abnormality. Mtb infection in adipose tissue causes mTOR/Akt-mediated inflammation and immune cell infiltration into adipose tissues, which is responsible for adipocyte hypertrophy, increased insulin sensitivity and may result in systemic glucose tolerance (Martinez et al., 2019). Interestingly, neutrophils in hyperglycemic subjects had reduced chemotaxis (Tater et al., 1987), phagocytic defect and reduced microbicidal activity (Chanchamroen et al., 2009).

It is apparent that a proper Th1-biased adaptive immune response is the major determinant of outcomes in human TB and in animal models of TB. Delayed priming of cell-mediated immunity appears to be one of the major reasons for higher susceptibility to tuberculosis in hyperglycemic mice (Vallerskog et al., 2010). IFNγ produced by Th1 cells is critical for protective immune response of the host while IL-2 is essential for the development and proliferation of Th1 and CD8+ cells. In streptozotocin-induced diabetes mellitus mice model, expression of Th1 cytokines and inducible nitric oxide synthase was found to be lower upon infection with Mtb (Yamashiro et al., 2005). However, in diabetic individuals, IFNγ secretion by immune cells was found to be not uniform. Stimulation of immune cells with mycobacterial antigens, either no difference (Stalenhoef et al., 2008) or lower (Meenakshi et al., 2016) or higher (Gan et al., 2014) secretion of IFN-γ was observed in DM patients compared to healthy controls. In other studies, hyperglycemia was found to be associated with suppression of innate and adaptive immune responses in TB-DM patients as absolute numbers of total T and B lymphocytes, CD8+ T lymphocytes, and NK cells were found to be lower (Wei et al., 2022). Experiments from mice model of infection suggest that an impaired innate response to initial infection with a resulting delay in the adaptive immune effector response is the key mechanism of susceptibility to tuberculosis in diabetes (Martinez and Kornfeld, 2014).

Diabetics with latent TB infection (LTBi) had increased inflammatory responses indicating that diabetic condition influences immune signaling to mycobacterial antigens (Ssekamatte et al., 2021; Masood et al., 2022). In a diet-induced type 2 diabetes mice model (that reflects the features of human T2D), a higher mortality rate, increased lung and liver bacilli burden and higher inflammatory lesions were observed as compared to non-diabetic control mice (Alim et al., 2017; Alim et al., 2019). These mice showed impaired macrophage anti-mycobactericidal function, higher inflammatory lesions and impaired cytokine kinetics (TNF-α, MCP-1, IL-12, IFN-γ) (Alim et al., 2017). In another study, streptozotocin-induced DM had delayed priming of adaptive immune response leading to impaired TB defence in mice (Vallerskog et al., 2010). Even latent TB patients with pre-diabetic conditions have diminished frequencies of antigen–specific Th1/Tc1 and Th17/Tc17 cells, indicating that pre-diabetic condition is associated with alterations of the immune response with compromised CD4+ and CD8+T cell function in latent TB cases (Kumar et al., 2017a). The reduction of innate and adaptive immunity in diabetic patients causes the severity of TB disease and might be involved in adverse effects of TB treatment outcome, increased morbidity and mortality during TB-DM (Workneh et al., 2016; Prada-Medina et al., 2017). Higher levels of pro-inflammatory cytokines were observed in TB-DM patients compared to non-diabetic patients before and throughout the anti-tuberculosis therapy (Kumar et al., 2019). TB-DM comorbidity is attributed to excessive and prolonged inflammation and organ damage and morbidity/mortality in pulmonary TB largely reflects the consequences of immune-mediated lung damage (Ravimohan et al., 2018). Hyperglycaemic patients are likely to develop cavitary lung lesions and poor treatment outcomes with higher post-treatment mortality (Williams et al., 2022). In fact, diabetic patients were found to develop more frequent lung cavities and parenchymal lesions upon chest X-ray (Chiang et al., 2014). diabetic patients with poor glycemic control are likely to be presented with atypical findings upon chest X-ray and thoracic CT scans, such as advanced extensive lesions (p < 0.001), more cavities (p < 0.001) and all-lobe involvement (Huang et al., 2017). Hyperglycemia in DM patients suggested to be a risk factor for pulmonary cavity formation and lobe lesions in patients with TB-DM which adds to TB pathology (Wei et al., 2022). Chronic renal failure is another risk factor for the infection progression of tuberculosis and chronic renal failure and dialysis increase the risk of TB infection by 6.9 to 52.5-fold compared to the general population (Hussein et al., 2003). Thus, both type 1 and type 2 diabetes decrease immune response against tuberculosis increasing the chance of organ pathology and disease susceptibility with complications of anti-TB treatment and death in TB.

## 4 Therapeutic management

Searching for strategies to prevent diabetic conditions is of utmost importance. Various factors, like pro-inflammatory cytokines, inflammation, reactive oxygen species, glyco/lipoxidation end products are shown to be involved in the pathogenesis of diabetic kidney disease, diabetic retinopathy, and on the progression of atherosclerosis and diabetes-associated non-alcoholic fatty liver disease (NAFLD). Mtb can act in a pleiotropic manner affecting adipose tissue signaling, inflammation, host metabolism (glucose and lipid) causing hyperglycaemia and insulin resistance that may lead to diabetes. Thus, strategies need to be devised to control/manipulate such factors during infection with Mtb to regulate diabetes and associated disorders in human. It has been shown that drugs used to treat tuberculosis can worsen the glycaemic control in diabetic patients. Overlapping toxicities should be considered when co-managing tuberculosis and diabetes. It is suggested that pyridoxine should be given with isoniazid during tuberculosis treatment in diabetic patients considering the risk of peripheral neuropathy (American Thoracic Society, CDC, Infectious Diseases Society of America, 2003). Treatment with rifampicin probably causes hyperglycaemia *via* interactions with oral hypoglycaemic drugs (Atkin et al., 1992; Niemi et al., 2001). Rifampicin causes early-phase hyperglycaemia with associated hyperinsulinaemia even in non-diabetic patients (Takasu et al., 1982; Waterhouse et al., 2005). Therefore, careful attention needs to be opted in delivering the anti-tuberculosis drugs in managing hyperglycaemia and diabetes. Also, therapeutics may be designed tox inhibit excess inflammation during tuberculosis. Since expression of MCP-1 was shown to be correlated with appearance of insulin resistance, management of MCP-1 expression during TB could serve as an important strategy to control type 2 diabetes. During infection, Mtb shifts the carbon source from glucose to fatty acids and utilizes glyoxylate cycle (Mukhopadhyay et al., 2012). Expression of the gating enzyme of glyoxylate cycle, isocitrate lyase (ICL) is upregulated during infection, and is important for survival during the persistent phase of infection. Thus, blocking of ICL could be important to tackle the Mtb infection and Mtb-associated systemic metabolic complications (Erol, 2008). One of the possible mechanisms to counter the manipulating effect of Mtb is to target its ability to modify host metabolism (glucose and lipid). It is thus important to target the secretory/effector proteins/molecules of Mtb (like ESAT-6 and lipases) that cause metabolic perturbation. Also, management of inflammation during TB could be another therapeutic measurement to control hyperglycaemia and type 2 diabetes. An interesting study indicates that binding of Mtb proteins specifically to 16~20 TLR2 LRR domain can trigger NF-κB signaling and TNF-α production in macrophages indicating the importance of such signaling in the regulation of pro-inflammation response which may lead to insulin resistance and type 2 diabetes (Bhat et al., 2012). Thus, the site of interaction on TLR2 can dictate the downstream signaling events leading to activation of NF-κB and pro-inflammation, which can be manipulated in the regulation of inflammatory signaling during TB diseases by designing appropriate drugs to specifically block TLR2 LRR 16~20-triggered signaling cascades. Another study indicates that cellular localization of Mtbhsp60 post interaction with TLRs can dictate the type of polarization in the innate immune responses in macrophages (Parveen et al., 2013). The results indicate that NF-κB and TNF-α activation by Mtbhsp60 was restricted predominantly to TLR4-triggered signaling. This information is useful in devising strategies to manipulate macrophage innate responses and block the excess pro-inflammatory signaling during TB which may be useful to tackle type 2 diabetes. Thus, therapeutic strategies targeting inflammation may be important for designing of effective treatment of diabetic complications during TB. Interestingly, a recent study highlights the protective efficacy of ESX-1-expressing BCG strains against Mtb infection in a diet-induced murine model of type 2 diabetes which is due to increased numbers and activation of antigen presenting cells in the lungs of diabetic mice (Sathkumara et al., 2020). Important strategies to stop development of TB might include, early diagnosis and early treatment adaptation, targeted vaccination programmes, screening for latent TB and diabetes among TB patients as well as improved diabetes management and prevention.

## 5 Conclusion and future perspective

TB-DM co-incidence is a major concern among tuberculosis patients as it complicates treatment course and disease outcome drastically. Various studies support that TB can influence the pathogenesis and incidence of diabetes (Mugusi et al., 1990; Magee et al., 2018). Occurrence of hyperglycaemia, insulin resistance and metabolic alterations during tuberculosis suggests the involvement of Mtb factors/proteins in development of pre-diabetic/diabetic conditions. Although various studies support that changes in immune responses/signaling cascades and host physiology during Mtb infection can induce hyperglycaemia that can stimulate pre-diabetic conditions, many of the important questions regarding host factors and signaling cascades that can influence occurrence of diabetes in TB patients are yet to be answered. It is very important to identify what Mtb factor is involved in development of hyperglycaemia and pre-diabetic/diabetic conditions which is crucial for the design of appropriate therapeutics to prevent this patho-physiological disorder during TB. Another important issue is the status of DM prevalence with respect to MDR/XDR TB which has to be seriously investigated. Also, studies need to be focused to understand the influence of dormancy/latency on TB-DM as diabetes and latent TB can be considered as predisposing factors for TB progression. Considering, the increasing cases of TB-DM, and the facts that TB-DM can enhance DM-linked complication pathways compared to DM alone (Prada-Medina et al., 2017), and also TB-DM co-occurrence can cause adverse effect on TB treatment and outcome of disease, the regular screening of TB patients for diabetes is important. Further, the host-directed therapy approach is important for TB-DM treatment to control immune-modulation and metabolic alterations that occurs during tuberculosis.

## Author contributions

MB — Conceptualization, Preparation of first draft and revision. PD — Writing and review. SG — Manuscript writing and editing. SM — Conceptualization, writing, review and finalization of the manuscript. All authors contributed to the article and approved the submitted version.
